# Differential dehydration effects on globular proteins and intrinsically disordered proteins during film formation

**DOI:** 10.1002/pro.3118

**Published:** 2017-02-07

**Authors:** Juliana Sakamoto Yoneda, Andew J. Miles, Ana Paula Ulian Araujo, B. A. Wallace

**Affiliations:** ^1^ Institute of Structural and Molecular Biology, Birkbeck College, University of LondonLondonUK; ^2^ Instituto de Física de São Carlos, Universidade de São PauloSão CarlosBrazil; ^3^ Present address: Institute of PhysicsUniversidade de São PauloSão PauloBrazil

**Keywords:** globular proteins, intrinsically disordered proteins, synchrotron radiation circular dichroism (SRCD) spectroscopy, dehydration, unfolding, folding, secondary structure

## Abstract

Globular proteins composed of different secondary structures and fold types were examined by synchrotron radiation circular dichroism spectroscopy to determine the effects of dehydration on their secondary structures. They exhibited only minor changes upon removal of bulk water during film formation, contrary to previously reported studies of proteins dehydrated by lyophilization (where substantial loss of helical structure and gain in sheet structure was detected). This near lack of conformational change observed for globular proteins contrasts with intrinsically disordered proteins (IDPs) dried in the same manner: the IDPs, which have almost completely unordered structures in solution, exhibited increased amounts of regular (mostly helical) secondary structures when dehydrated, suggesting formation of new intra‐protein hydrogen bonds replacing solvent‐protein hydrogen bonds, in a process which may mimic interactions that occur when IDPs bind to partner molecules. This study has thus shown that the secondary structures of globular and intrinsically disordered proteins behave very differently upon dehydration, and that films are a potentially useful format for examining dehydrated soluble proteins and assessing IDPs structures.

## Introduction

Native globular protein structures, which usually consist of significant amounts of regular helical, sheet, and turn secondary structures, are driven and/or stabilised by hydrogen bonds, electrostatic forces, van de Waals, and hydrophobic interactions. Their three‐dimensional structures generally also include significant numbers of partially‐ and fully‐ ordered buried water molecules^1^, ^2^ present in internal cavities, which can contribute to the stability of the protein.^3^ Canonical protein secondary structures (helices, sheets, and turns) tend to satisfy their hydrogen bonding potentials within the secondary structural elements rather than with surrounding water molecules; statistical analyses have shown that buried waters are most often associated with polypeptide backbone atoms not participating in regular secondary structural elements.^1^ Globular proteins also include many buried residues that are not in contact with any solvent molecules. In contrast, intrinsically disordered proteins (IDPs) exhibit limited amounts of regular secondary structures, have minimally ordered tertiary structures in solution, and tend to have most residues exposed to solvent, involving both side chain and backbone interactions with water molecules.^4^ Consequently they have extensive interactions with aqueous solvent throughout their length.

The influence of removing the water on the structures of both types of proteins is of interest for two quite disparate reasons: (1) the creation and stability of pharmaceutical formulations, and (2) the roles of water in intrinsically disordered protein structures and their intermolecular and intramolecular interactions.

One of the impediments to using proteins in pharmaceutical formulations has been their inherent instability upon dehydration, as proteins are sensitive to a variety of physical and chemical degradative processes, which are either facilitated or directly mediated by loss of water.^5^, ^6^ Common bioindustrial dehydration processes are lyophilization or freeze‐drying, spray‐drying^7^, ^8^ and microglassification,^9^ which may have different effects on the protein integrity. Fourier‐transform infrared (FTIR), Raman and solid state nuclear magnetic resonance (NMR) spectroscopic investigations of several soluble globular proteins have indicated that protein denaturation may be induced by lyophilization,^6^, ^10^, ^11^ spray‐drying,^12^ and precipitation with organic solvents followed by drying.^10^ These procedures in general resulted in a significant loss of native secondary structures contents. For example, following lyophilization, the proteins studied exhibited significantly increased the β‐sheet contents and decreased the α‐helix contents, although the magnitudes of the effects varied amongst the proteins.^10^

By comparison, a study^13^ on the effect of dehydration on MEG‐14, an intrinsically disordered protein (IDP), using synchrotron radiation circular dichroism (SRCD) spectroscopy, suggested that the MEG‐14 protein gained regular (mostly helical) structure upon dehydration into films. IDPs are a class of proteins that generally exhibit little regular secondary structure in solution.^4^ It was suggested^13^ that dehydration could be a possible way of mimicking their interactions/conformational changes upon binding partner molecules (a process which may involve partial dehydration upon exclusion of water molecules in the complexes). Because dehydration in that study was achieved in a physical process different from lyophilization, and because the result contrasts with the previous FTIR studies on globular proteins that showed a decrease in helical content, or increase in sheet (which could be a manifestation of aggregation) when dehydrated, in this study we examined both globular and intrinsically disordered proteins in which the dehydrated state was produced by the same process of drying into films. SRCD spectroscopy was used to monitor the effect of dehydration. The aim was to evaluate if the differential effects observed for globular and IDPs are due to the method used to monitor the structures or due to the nature of the proteins examined. The measurements of dehydrated samples were then compared with SRCD spectra of the IDPs after rehydration to identify if the effects of dehydration were reversible.

The advantage of using SRCD spectroscopy rather than conventional CD spectroscopy in this study was because the high light flux of the synchrotron source enables light penetration into (and thus measurements) of films that absorb light strongly.^14^ In addition, the shorter wavelength data measurable with SRCD spectroscopy are particularly valuable for detecting disordered structures in proteins, as the characteristic peaks for these features are located at wavelengths below 190 nm.^14^ It is noteworthy that the presence of water in aqueous solution samples normally limits the wavelengths measurable (even by SRCD spectroscopy) to around 170 nm, due to the strong water absorption peak near this wavelength. However, measurements in dehydrated films allow acquisition of data to considerably shorter wavelengths, and, most importantly, provides a means of monitoring the water content in the dehydrated samples.

## Results

**Figure 1:**
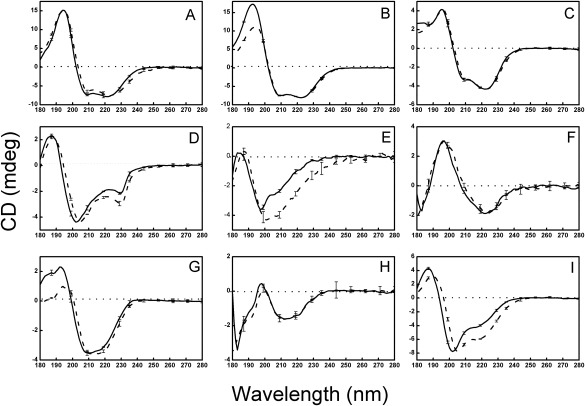
CD Spectra of Globular Soluble Proteins. CD spectra of soluble globular proteins representing the four CATH classes (mostly helical: panels A, B, and C; mostly sheet: panels D, E, and F; mixed helix/sheet: panels G and H; and irregular: panel I). Solid lines represent solution spectra, while dashed lines are from films. The error bars indicate one standard deviation in three repeated measurements. The proteins are A: hemoglobin, B: myoglobin, C: cytochrome C, D: α‐chymotrypsin, E: elastase, F: concanavalin A, G: ribonuclease A, H: carbonic anhydrase II, I: aprotinin.

CD spectra of solutions and films were collected for soluble globular proteins included representatives of the four main CATH classes^15^ (Fig. 1, Table 1): 1 [mostly helical] (hemoglobin, myoglobin, and cytochrome c), 2 [mostly sheet] (α‐chymotrypsin, elastase and concanavalin A), 3 [mixed helix/sheet structures] (ribonuclease A, carbonic anhydrase II) and 4 [mostly unordered] (aprotinin). In general, the calculated helical and sheet secondary structures derived from the CD spectra corresponded closely to the secondary structures as found in the crystal structures (Table 1).

**Table 1:** Calculated Secondary Structures of the Soluble Globular Proteins

Protein		α‐helix	β‐sheet	turn	other	NRMSD	CATH
HBN	S	73 ± 3	2 ± 1	9 ± 0	15 ± 3	0.020	
	F	70 ± 5	2 ± 1	9 ± 1	17 ± 6	0.060	
	PDB	77	0		23		1.10.490.10
MBN	S	73 ± 2	1 ± 1	10 ± 0	17 ± 3	0.011	
	F	65 ± 7	4 ± 4	11 ± 1	22 ± 6	0.038	
	PDB	74	0		26		1.10.490.10
CYC	S	38 ± 1	14 ± 4	13 ± 1	35 ± 2	0.033	
	F	37 ± 2	15 ± 3	12 ± 1	35 ± 2	0.042	
	PDB	37	0		63		1.10.760.10
CHM	S	20 ± 0	21 ± 1	15 ± 0	43 ± 1	0.051	
	F	33 ± 4	11 ± 1	17 ± 1	40 ± 4	0.048	
	PDB	12	32		56		2.40.10.10
ELA	S	9 ± 2	32 ± 3	15 ± 1	44 ± 0	0.072	
	F	15 ± 1	27 ± 1	15 ± 0	43 ± 1	0.068	
	PDB	10	30		60		2.40.10.10
CON	S	9 ± 3	40 ± 2	11 ± 1	38 ± 1	0.057	
	F	8 ± 3	41 ± 2	12 ± 1	38 ± 1	0.108	
	PDB	4	46		50		2.60.120.200
RNS	S	23 ± 1	29 ± 1	12 ± 1	35 ± 1	0.040	
	F	18 ± 1	31 ± 1	12 ± 1	38 ± 1	0.056	3.10.130.10
	PDB	21	33		46		
CAH	S	11 ± 3	35 ± 2	12 ± 0	40 ± 1	0.085	
	F	12 ± 3	35 ± 2	13 ± 1	41 ± 2	0.115	
	PDB	16	29		55		3.10.200.10
APO	S	23 ± 1	23 ± 1	11 ± 2	44 ± 2	0.019	
	F	45 ± 8	11 ± 7	13 ± 1	33 ± 6	0.125	
	PDB	21	24		55		4.10.410.10

Secondary structures of the proteins in solution (S), in films (F), and calculated from PDB files using the DSSP algorithm. Their CATH classification is also listed. The values determined from the solutions and films are the average calculations derived from 3 different algorithms, with the ± values indicating the S.D. between the methods, and the NRMSD is a goodness‐of‐fit parameter. The protein names are abbreviated as: HBN, hemoglobin; MBN, myoglobin; CYC, cytochrome C; CHM, alpha‐chymotrypsin; ELA, elastase; CON, concanavalin A; RNS, ribonuclease A; CAH, carbonic anhydrase II; and APO, bovine trypsin inhibitor (aprotinin).

For the globular protein films, the principal observation was that the spectra for the proteins in the two physical forms (solution and film) (Fig. 1) were very similar up to the wavelength where the HT (pseudo‐absorbance) indicated the light penetration into the film was too low (Supporting Information Fig. S1) to make accurate measurements. Some spectra included a slight red‐shift in the peak wavelengths upon dehydration, which was likely to be a result of a change in the dielectric constant adjacent to some of the peptide bonds resulting from differences in energy between ground and excited states, as has been seen previously when proteins were subjected to solvent dielectric changes.^16^ In most cases the calculated secondary structure content was not substantially different for the same protein in the two states (Table 1). Only in a few globular protein cases (α‐chymotrypsin, elastase, and aprotinin), did the spectra in solution and films differ to any significant extent: they all tended to exhibit an increase in the peak around 224 nm, resulting in a slight increase in the calculated helical content for the films. It is noteworthy that in each of these cases, the protein has a substantial irregular (not helical, not sheet) secondary structure content in solution (indeed aprotinin is in the CATH class 4 — indicative of “irregular” or little secondary structure^15^).

To determine if the observations on the films were associated with the specific nature of the substrate used, films were produced on plates formed from two different materials (quartz and calcium fluoride) with different wetting (contact angle) properties; the resulting spectra were very similar (Supporting Information Fig. S2), suggesting that the interactions with the substrates were not the main reason for the similarities between the solutions and films.

**Figure 2:**
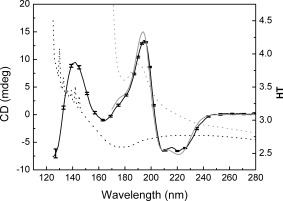
SRCD and HT Spectra of Hemoglobin in Solution and Films. These spectra demonstrate that the film samples have little or no water remaining, due to the lack of a peak in the HT spectrum (dotted black line) around 170 nm. This lack of water also enables the film SRCD spectrum (solid black line) to be measured to shorter wavelengths (∼125 nm). The series of sharp peaks in the HT spectrum below ∼140 nm are due to absorbance of the nitrogen gas flushing the sample chamber. By comparison, the HT spectrum of the solution (dotted grey line) exceeds the maximum cutoff value of 5.0 at all wavelengths below 172 nm due to edge of the large water absorption band, which limits the lowest wavelength measurable in the SRCD spectrum (solid grey line) to ∼170 nm.

**Figure 3:**
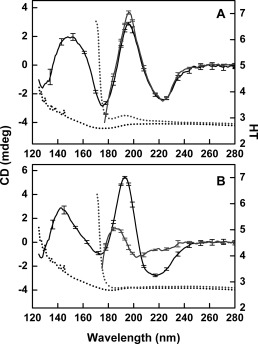
Effect of Rehydration on the SRCD Spectra of Protein Films. Black solid lines are dehydrated films, gray solid lines are resolubilized films, and dashed lines are HT spectra in the same respective colors as Figure 2. (A) Concanavalin A (globular) and (B) β‐synuclein (IDP).

To determine if the water had been effectively removed from the films during the dehydration process, samples were measured using SRCD spectroscopy, while the HT spectra were collected at the same time (Fig. 2). In the solution sample, the beginning of the large peak due to water [located at ∼165 nm] is visible in the HT curve, starting at around 180 nm, so the lowest wavelengths measurable (i.e., the cutoff) in the SRCD spectra were ∼170 nm. From the HT spectra of the films, however, it could be seen that the water peak at ∼165 nm is essentially completely missing, with the cutoff occurring at around 126 nm (which is essentially due to the absorbance of the substrate). This indicated that all or most of the bulk water had been removed during the process of film formation. Note that these measurements required SRCD instrumentation as the water peak observed (or missing) is at a much shorter wavelength than the instrumental cutoff (∼180 nm) for CD spectra. Upon rehydration of the films, the secondary structure was retained (Fig. 3a).

The results obtained in this study on dehydrated globular proteins, are quite different from those reported in earlier FTIR studies of lyophilized proteins.^6^, ^10^ To determine if these different observations were technique‐ or sample‐ associated, it was possible to compare several of the proteins that were used in both the present study and one of the previous FTIR studies of lyophilized proteins: aprotinin (also called BPTI), ribonuclease A, myoglobin and cytochrome C. In general, the helical secondary structures calculated for solutions of those proteins from the CD and FTIR spectroscopic studies were quite similar (Supporting Information Table S1). However, the FTIR lyophilization study indicated most proteins decreased in helix content while their sheet content increased^10^ (Supporting Information Table S1) upon dehydration. In contrast, in this study, most globular proteins produced very similar spectra (Fig. 1) and calculated secondary structures (Supporting Information Table S1) for both solutions and films. The reason for the differences between this study and the previous studies could be the process of dehydration: during lyophilization, protein aggregation may have occurred, which often produces an apparent increase in beta sheet, such as that associated with amyloid formation. The dehydrated film samples examined by CD spectroscopy may have retained some water content (most likely intramolecular waters which are tightly bound and not removed by the process) although the simultaneous HT measurements on the samples described above indicate the water content remaining is very small (Fig. 2). This retained water may contribute to the retention, rather than collapse, of the regular secondary structures present in the proteins. Furthermore, as nearly identical spectra of the films were obtained when the proteins were dried onto the two types of plates with different wetting properties (silica quartz, which is relatively hydrophilic, and calcium fluoride which produces much larger contact angles, thus indicating it provides a more hydrophobic surface for interaction with the protein) (Supporting Information Fig. S2), it was possible to rule out the interactions with the substrates as contributing to any folding/unfolding effects.

**Figure 4:**
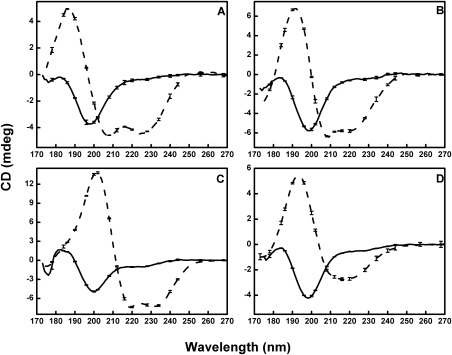
SRCD Spectra of Intrinsically Disordered Proteins. SRCD spectra of intrinsically disordered proteins: (A) MEG‐14, (B) β‐synuclein, (C) SHERP, and (D) α‐synuclein, all of which are almost entirely unstructured (irregular secondary structure) in solution (solid lines). Dashed lines are from films, where the proteins have much more regular (helical) secondary structures.

The most dramatic finding in this study, however, was the differential effects of dehydration on globular and IDP proteins. While there was little change in the secondary structures of globular proteins, when water was removed from the samples, IDPs (which exhibit little persistent regular structure in solution) tended to produce vastly different spectra upon dehydration (Fig. 4), consistent with a large gain in the amount of regular secondary structure (primarity α‐helix) as they underwent the process of dehydration (Table 2). The increase in the amount of helical structure was entirely reversible if the films were rehydrated [Fig. 3b]. Hence, it was not simply an irreversible aggregation effect. This ordering/unordering effect is in line with the previous observations on the IDP MEG‐14,^13^ where a substantial increase in helix content was observed upon dehydration.

**Table 2:** Secondary Structures of the Intrinsically Disordered Proteins

Protein		α‐helix	β‐sheet	turn	other	NRMSD
MEG	S	4 ± 2	23 ± 2	15 ± 1	51 ± 8	0.036
	F	32 ± 6	11 ± 1	14 ± 0	41 ± 6	0.182
						
BSN	S	3 ± 1	10 ± 4	9 ± 4	75 ± 6	0.042
	F	37 ± 2	13 ± 1	14 ± 1	36 ± 1	0.066
						
SHP	S	7 ± 2	14 ± 2	11 ± 2	68 ± 3	0.073
	F	69 ± 4	4 ± 2	11 ± 4	17 ± 6	0.018
						
ASN	S	4 ± 2	17 ± 4	13 ± 3	62 ± 4	0.035
	F	35 ± 1	25 ± 2	18 ± 4	22 ± 5	0.045

Secondary structures of the intrinsically disordered proteins in solution (S), in films (F). The values determined from the solutions and films are the average calculations derived from three different algorithms, with the ± values indicating the S.D. between the methods, and the NRMSD as a goodness‐of‐fit parameter. The protein names are abbreviated as: MEG, MEG‐14; BSN, β‐synuclein; SHP, SHERP; ASN, α‐synuclein.

It is noticeable that the most substantial differences between solution/film samples in the soluble globular proteins were for aprotinin (APO) and elastase (ELA), proteins chosen because they have less regular secondary structure (Table 1) and more irregular structure than the other globular proteins examined. This is consistent with the observations that the IDPs change their structure dramatically. Although the APO and ELA proteins are sufficiently well ordered to form crystal structures, they do represent examples of globular proteins that contain significant amounts of disordered residues. From the present study, it appears that these proteins (which have intrinsically disordered regions (IDRs)^17^) may behave to a lesser, but detectable, extent as do the fully IDPs by becoming more regular when dehydrated.

## Discussion

In this study, it was shown that soluble globular proteins do not significantly change their conformations upon dehydration into films. These results differ considerably from previous studies of proteins dehydrated by lyophilization^6^. They suggest that the different types of dehydration processes (lyophilization and film formation) produce different effects on the structures of soluble globular proteins, with proteins retaining near‐native structures upon film formation, but undergoing significant conformational changes upon lyophilization. These differing observations could be a consequence of the use of different proteins in the different studies, the manner in which the proteins associate during the drying, the interactions of the proteins with the substrates in films, and/or the amounts and locations of water molecules remaining in the samples. Direct comparisons of the effects of the dehydration procedures could be made for some of the proteins examined in this study and the lyophilized proteins studied previously by FTIR spectroscopy,^6^ because a number of the same proteins were used in both studies. Those direct comparisons (Supporting Information Table S1) suggest that the two processes have different effects on the protein structures, and the differences observed are not because different proteins were used in different studies, nor were they apparently due to the use of different spectroscopic methods in the two studies, as the cognate solution structures in both studies tended to be quite similar. Furthermore, since the studies examined the same protein on two different materials with different wetting properties, the nature of the film interactions with the solid substrates could be eliminated as the source of the retention of the native‐like folded structures. It was not possible to detect if any minor amount of water was retained in the films (although all bulk water was demonstrated to have been removed by virtue of the lack of a water peak in the low wavelength HT spectra), so it may be that a few buried waters remain in the films, helping the proteins to retain their native‐like structures; perhaps during the process of lyophilization even the internal water molecules are stripped away, contributing to the differences seen. A final possibility is that some of the differences could be more apparent than real, due to the nature of the FTIR measurements: Given the difficulty in precisely removing the contribution of water solvent baselines during the processing of the FTIR data, some apparent changes in the lyophilization studies could be due to spectral artifacts in those studies. Such effects would vary with remaining water content (something very difficult to measure); the presence of small amounts of remaining water, however, would not affect the CD spectra, as water molecules do not contribute to the chiral signals measured by this method.

In contrast to the dehydration studies on globular proteins in films, dehydration of IDPs produced an increase in regular secondary structures in dried films, which was a completely different behavior than that seen for the globular proteins, even though both types of proteins were dehydrated in the same manner. IDPs gained order in the form of increased canonical secondary structure contents (primarily helix), whereas globular proteins tended to retain very similar secondary structures. This suggests that removal of water in dehydrated films of soluble proteins does not cause the unfolding or refolding of the proteins, but that removal of the water in IDPs does. We speculate that this is because in IDPs, the peptides form intermolecular hydrogen bonds with the water solvent molecules to the exclusion of intramolecular hydrogen bonds within the protein, so they don't exhibit significant amounts of regular helical or sheet secondary structures. The removal of the surrounding water in the IDPs enables the formation of intramolecular hydrogen bonds and the formation of helices (and to a lesser extent) sheets. However, these changes are transient and can be broken when the bulk water is restored. This type of behavior may mimic the types of transient interactions involving intermolecular hydrogen bonding between IDPs and their partner molecules, which has been suggested as a basis for their promiscuous interactions which have been proposed to be related to their roles in regulation and intermolecular interaction processes.

## Materials and Methods

Concanavalin A (*Canavalia ensiformis*) [CON], carbonic anhydrase II (*Bos taurus*) [CAH], elastase (*Sus scrofa*) [ELA], bovine trypsin inhibitor (aprotinin) [APO], α‐chymotrypsin (*Bos taurus*) [CHM], cytochrome C (*Equus caballus*) [CYC], were purchased from Sigma. Ribonuclease A (*Bos taurus*) [RNS], myoglobin (*Equus caballus*) [MBN], and hemoglobin (*Bos taurus*) [HBN] were from Calbiochem. Human β‐synuclein [BSN] and human α‐synuclein [ASN] were purchased from Sigma‐Aldrich (United Kingdom). Micro‐exon gene protein 14 (MEG‐14) [MEG] was prepared as previously described,^13^ and the Small Hydrophilic Endoplasmic Reticulum‐associated Protein (SHERP) [SHP] was prepared as described in Moore *et al*.^18^

For the films, an aliquot (10–20 μl) of each protein (at a concentration of 1.0–3.0 mg/mL) in 10 mM sodium phosphate buffer, pH 7.4, was deposited on the surface of a quartz Suprasil (Hellma UK, Ltd.) plate or a calcium fluoride plate (Hellma Jena, Germany) to form a dried film with an area about 1.0 cm^2^. For spectra collected to very low wavelengths (<155 nm), calcium fluoride plates were used; all other film samples described were deposited on quartz plates. The plates were placed on a flat surface for 20 to 30 min at room temperature to form the film by the slow evaporation of the solvent and then placed under vacuum for four hours to remove any residual solvent. Subsequently, each was placed in a specially‐designed cell holder to prevent water reabsorption. The sealed cell holder contained another Suprasil (or calcium fluoride) plate separated from the protein‐containing plate by a spacer of several mm^19^ and could be rotated without moving the area of the film (∼ 5 mm diameter) that was in the beam.^20^ Each sample was rotated in 90° increments (to ensure there was no contribution of linear dichroism to the spectrum) (Supporting Information Fig. S3). Three replicate spectra were obtained at each of the 90° rotation positions, making a total of 12 sample spectra, which were then averaged to produce the average spectrum. From this the final net spectrum was produced by subtracting a baseline, that was also the average of 12 raw spectra produced in the same way, but measured for the plates alone (without any sample). The solutions of the same proteins were examined using standard demountable Suprasil (Hellma UK, Ltd.) quartz cells with pathlengths of 0.01 cm. Three scans were collected for the protein solutions and baselines (10 mM sodium phosphate, pH 7.4). Film rehydration was achieved by placing 3 µL of the appropriate buffer onto the center of the film and then placing a plate with a 0.005 cm pathlength well on top and allowing the protein to resolubilize.

SRCD spectra were obtained at the Soleil (France), ISA (Aarhus, Denmark) or ANKA (Karlsruhe, Germany) synchtrotron beamlines. The same instrument parameters (1 nm interval, 25^o^C) were used with dwell times of 1.2 s, 2 s, and 3 s, respectively, at the 3 different synchrotrons, to obtain the spectra for the solutions and the films. The samples on calcium fluoride plates were measured over the wavelength range from 280 to 125 nm, while, in general, the other spectra were measured over the wavelength range from 280 to ∼180 nm. High Tension (HT) spectra (Supporting Information Fig. S1) were measured at the same time as the SRCD spectra to demonstrate the level of light penetration into the samples, thereby indicating the wavelength cutoff of the spectra.^21^ The CD signal is the absorbance of left circularly polarized light minus the absorbance of right circularly polarized light and is independent of the total photon flux through the sample. However photomultiplier detectors used in CD and SRCD spectroscopy require a certain level of input light to make accurate measurements. At wavelengths where the sample absorption is low, the photon flux reaching the detector is adequate. At wavelengths where the absorption of the sample is high, the signal is maintained by applying an external current which is proportional to the decrease in light flux reaching the detector and therefore proportional to the absorbance of the sample. The HT signal is a measure of this current and therefore a measure of the pseudoabsorbance of the sample. The cutoff wavelength is determined for each instrument^21^ as the wavelength corresponding to maximum HT value where enough light penetrates so that accurate data can be measured. For the spectra shown in Supporting Information Figure S1, the cutoff wavelength was determined to occur when the HT value was greater 500 mV.

Conventional CD spectra, primarily obtained as preliminary tests of sample conditions for the SRCD measurements, were made using an Aviv 430 instrument (Aviv Biomedical, Lakewood, NJ USA) over the wavelength range from 280 to 180 nm using wavelength steps of 1.0 nm, averaging time of 1 s, settling times of 0.33 s, and bandwidth of 1.0 nm. Three scans were collected for both the sample and baselines.

The low wavelength (<155 nm) films were measured at eight different rotation positions of the plate (in 45 degree intervals) with 1 scan at each position, to assess whether there was any linear dichroism contribution to the spectra, which would have resulted had there been any orientation/alignment of molecules within the films. For the rehydration experiments, data were collected from 280 to 170 nm on beamline CD1 at the ISA beamline, at 20°C using a step size of 1 nm and an averaging time of 2 s.

The spectra were processed using CDTool software,^22^ and for the solutions, scaled to delta epsilon values using mean residue weights of: hemoglobin (108.6), myoglobin (111.5), cytochrome C (113.6), α‐chymotrypsin (105.2), elastase (108.4), concanavalin A (108.7), ribonuclease A (111.3), carbonic anhydrase (112.3), and aprotinin (119.6). The protein concentrations of the solutions (∼1 mg/mL) were determined using a Nanodrop spectrophotometer (measurements in triplicate), with extinction coefficients calculated using EXPASY.^23^ For the films, the delta epsilon values were determined using the scale factor method,^24^ because the amount of deposited material actually in the area subtended by the beam could otherwise not be quantified. This method involves first scaling the spectral magnitudes so that they are roughly in the expected range of delta epsilon values for proteins, then iteratively using a fine multiplication factor [small increments (± 0.1)] to carry out a series of secondary structure analyses using the CONTIN algorithm, until the goodness‐of‐fit parameter (NRMSD) is minimized.

Secondary structure analyses were undertaken using the DichroWeb server^25^ with the ContinLL,^26^ Selcon3,^27^ and CDSSTR^28^ algorithms and the SP175 reference data set.^29^ The values derived from the 3 algorithms were averaged, and the ± values reported are the standard deviations of the results from the three methods. For Supporting Information Table S1, the algorithm producing the closest match to the crystal structure was used. The NRMSD value is a goodness‐of‐fit parameter calculated for the ContinLL method, indicating correspondence between calculated and measured spectra.^30^

Secondary structures derived from the corresponding crystal structures were determined using the DSSP algorithm^31^ available on the 2Struc server^32^ based on the following PDB codes: hemoglobin (1hda), myoglobin (1ymb), cytochrome C (1crc), α‐chymotrypsin (5cha), elastase (3est), concanavalin A (1nls), ribonuclease A (3rn3), carbonic anhydrase II (1v9e), and aprotinin (5pti). Their CATH classifications were obtained from the CATH server.^15^

## Data Deposition

The CD spectral and metadata for the solutions and dehydrated films have been deposited in the Protein Circular Dichroism Data Bank (PCDDB)^33^ under ID codes CD0005919000 to CD0005944000, and will be released upon publication.

## Supporting information

Supporting Information.
