# Supplementary material for: Differential dehydration effects on globular proteins and intrinsically disordered proteins during film formation
Source: Protein Sci. 2017 Feb 7;26(4):718–26. doi: 10.1002/pro.3118 (PMC5368061; doi:10.1002/pro.3118)
Supplement: Supplementary file 1 — Supporting Information. [file PRO-26-718-s001.docx]

**Differential Dehydration Effects on Globular Proteins and Intrinsically Disordered Proteins during Film Formation**

Juliana Sakamoto Yoneda^1,2,+^, Andew J. Miles^1^, Ana Paula Ulian de Araujo^2^,

and B.A. Wallace^1,*^

**Supplementary Material**

**Table S1. Comparison of secondary structures** of MBN, CYC, RNS, and APO in solution calculated from CD (S-CD) and from FTIR (S-FTIR) data, and in the dehydrated state: (films) using CD (D-CD) and (lyophilized) using FTIR (D-FTIR). The FTIR values are from ref 10. [note that APO – this work, is the same protein that is designated BPTI in ref 10]. delta-h = |%PDB helix-%calc helix|, delta-s=|%PDB sheet-%calc sheet|, change-H=(Shelix-Dhelix), change-S=(Ssheet-Dsheet).

| **protein** |  | **Helix** | **Sheet** | **delta-h** | **delta-s** | **change-H** | **change-S** |
| --- | --- | --- | --- | --- | --- | --- | --- |
| MBN | PDB | 74 | 0 |  |  |  |  |
|  | S-CD | 76 | 0 | 2 | 0 |  |  |
|  | D-CD | 74 | 1 | 0 | 1 | -2 | +1 |
|  | S-FTIR | 53 | 2 | 21 | 2 |  |  |
|  | D-FTIR | 28 | 25 | 46 | 25 | -25 | +23 |
|  |  |  |  |  |  |  |  |
| CYC | PDB | 37 | 0 |  |  |  |  |
|  | S-CD | 37 | 12 | 0 | 12 |  |  |
|  | D-CD | 40 | 14 | 3 | 14 | +3 | +2 |
|  | S-FTIR | 36 | 3 | 5 | 3 |  |  |
|  | D-FTIR | 23 | 35 | 18 | 35 | -13 | +32 |
|  |  |  |  |  |  |  |  |
| APO | PDB | 21 | 24 |  |  |  |  |
|  | S-CD | 21 | 23 | 0 | 1 |  |  |
|  | D-CD | 37 | 20 | 16 | 4 | +16 | -3 |
|  | S-FTIR | 21 | 36 | 0 | 12 |  |  |
|  | D-FTIR | 5 | 55 | 16 | 31 | -16 | +19 |
|  |  |  |  |  |  |  |  |
| RNS | PDB | 21 | 33 |  |  |  |  |
|  | S-CD | 22 | 31 | 1 | 2 |  |  |
|  | D-CD | 17 | 32 | 4 | 1 | -5 | +1 |
|  | S-FTIR | 23 | 45 | 2 | 12 |  |  |
|  | D-FTIR | 18 | 66 | 3 | 33 | -5 | +21 |

**Figure S1: CD and HT Spectra for Globular Proteins.**

The CD spectra from Figure 1 (black solid lines for solutions, red solid lines for films) are plotted with their respective HT spectra overlaid (black and red dashed lines), to show the different absorbances of the solutions and films. The arrows at the bottom of each panel indicate where the effective cutoff wavelengths (HT >500 mV) are for each spectrum. Panels in which no arrow is shown mean that neither sample type exceed the cutoff value at any wavelength.

**Figure S2: Examples of Dehydrated Samples Deposited on Different Types of Substrates.**


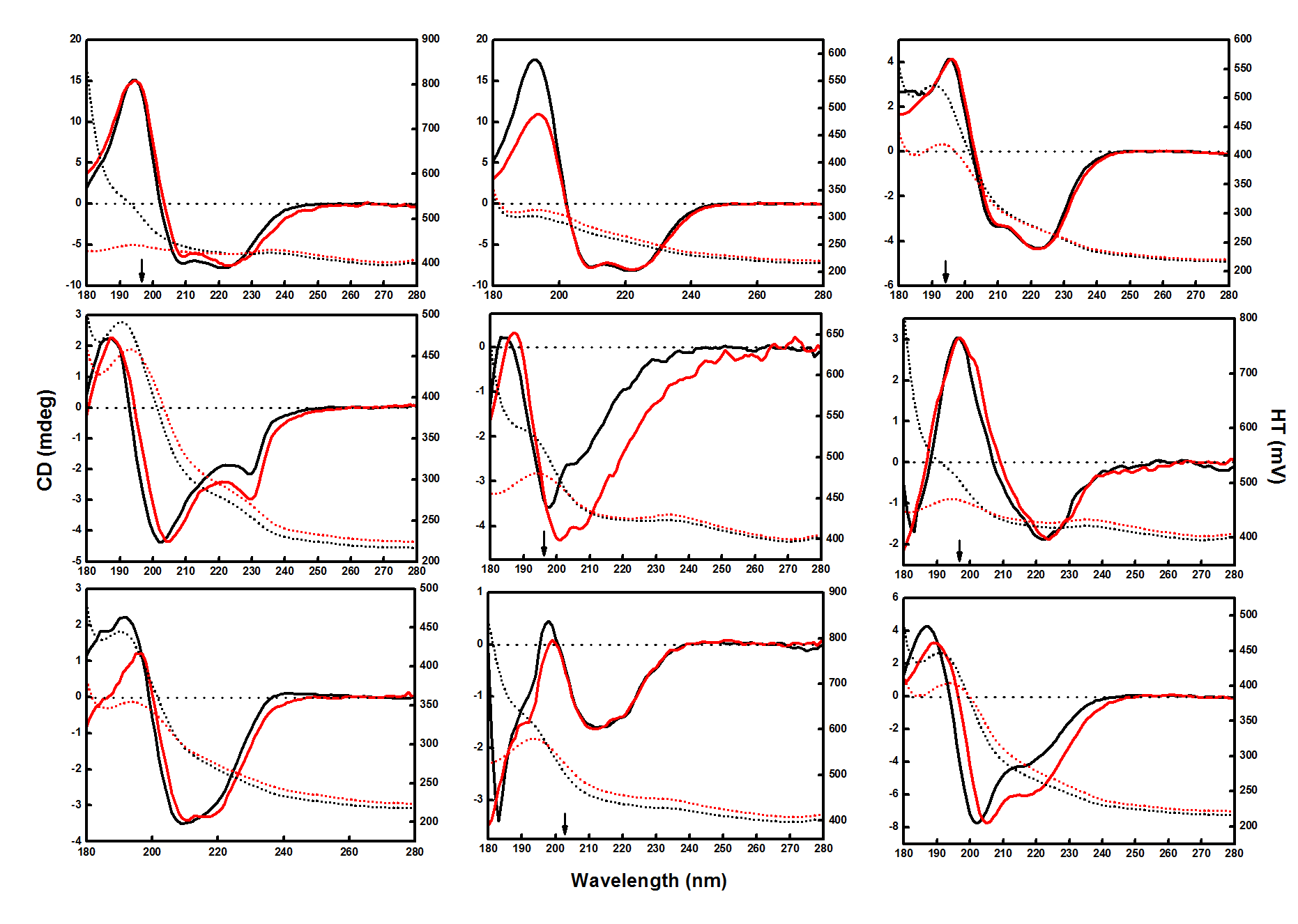


These spectra show there is little difference associated with different substrates used for making films. In each case, the black spectrum is the dehydrated protein on a silica quartz plate, and the blue spectrum is the dehydrated protein on a calcium fluoride plate. Examples are for A) the globular protein cytochrome c, and B) the IDP protein β-synuclein.


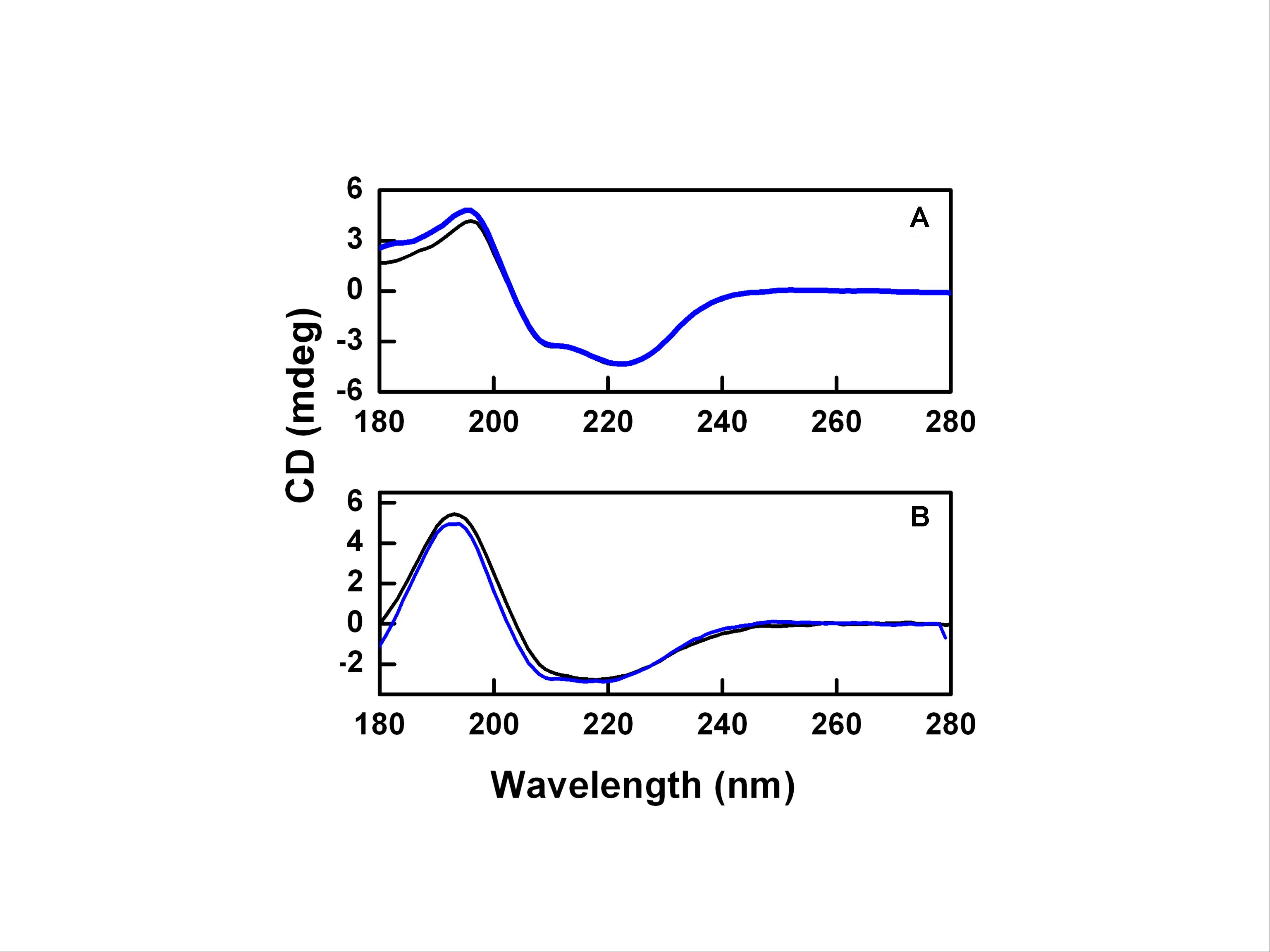


**Figure S3: The Effects of Rotating the Films Perpendicular to the Beam.**

These provide evidence that the measured SRCD spectra do not include any significant linear dichroism signal (i.e. they are not oriented). Each plot is the average of 4 successive 90 degree rotations, with the error bars showing the variation (1 s.d.) between the spectra for: A) concanavalin A, and B) β-synuclein.
